# Association between parental guilt and oral health problems in preschool children: a hierarchical approach

**DOI:** 10.1186/1471-2458-14-854

**Published:** 2014-08-16

**Authors:** Monalisa Cesarino Gomes, Marayza Alves Clementino, Tassia Cristina de Almeida Pinto-Sarmento, Carolina Castro Martins, Ana Flávia Granville-Garcia, Saul Martins Paiva

**Affiliations:** Postgraduate Program in Dentistry, State University of Paraiba (UEPB), 1325/410 Capitão João Alves de Lira, 58428-800 Campina Grande, PB Brazil; Department of Pediatric Dentistry and Orthodontic, Federal University of Minas Gerais (UFMG), Belo Horizonte, MG Brazil

**Keywords:** Guilt, Dental caries, Tooth injuries, Preschool child

## Abstract

**Background:**

Dental caries and traumatic dental injury (TDI) can play an important role in the emergence of parental guilt, since parents feel responsible for their child’s health. The aim of the present study was to evaluate the influence of oral health problems among preschool children on parental guilt.

**Methods:**

A preschool-based, cross-sectional study was carried out with 832 preschool children between three and five years of age in the city of Campina Grande, Brazil. Parents/caregivers answered the Brazilian version of the Early Childhood Oral Health Impact Scale (B-ECOHIS). The item "parental guilt" was the dependent variable. Questionnaires addressing socio-demographic variables (child’s sex, child’s age, parent’s/caregiver’s age, mother’s schooling, type of preschool and household income), history of toothache and health perceptions (general and oral) were also administered. Clinical exams for dental caries and TDI were performed by three dentists who had undergone a training and calibration exercise (Kappa: 0.85-0.90). Poisson hierarchical regression was used to determine the significance of associations between parental guilt and oral health problems (α = 5%). The multivariate model was carried out on three levels using a hierarchical approach from distal to proximal determinants: 1) socio-demographic aspects; 2) health perceptions; and 3) oral health problems.

**Results:**

The frequency of parental guilt was 22.8%. The following variables were significantly associated with parental guilt: parental perception of child’s oral health as poor (PR = 2.010; 95% CI: 1.502-2.688), history of toothache (PR = 2.344; 95% CI: 1.755-3.130), cavitated lesions (PR = 2.002; 95% CI: 1.388-2.887), avulsion/luxation (PR = 2.029; 95% CI: 1.141-3.610) and tooth discoloration (PR = 1.540; 95% CI: 1.169-2.028).

**Conclusion:**

Based on the present findings, parental guilt increases with the occurrence of oral health problems that require treatment, such as dental caries and TDI of greater severity. Parental perceptions of poor oral health in their children and history of toothache were predictors of greater feelings of parental guilt.

## Background

Despite advances in the prevention and treatment of oral health problems, high prevalence rates of dental caries [1, 2] and traumatic dental injury (TDI) [3, 4] continue to be found among preschool children. These conditions can have a negative impact on the oral health-related quality of life (OHRQoL) of the family [5–7], since parents feel responsible for their child’s oral health [8]. Indeed, parents play an important role in the oral health status of children and in seeking dental care [9, 10] and therefore tend to express feelings of guilt when their child exhibits oral health problems and/or treatment needs [5, 11]. Thus, parental guilt can be defined as an emotional state aroused by actions or intentions that are perceived as incorrect [12].

A number of factors are considered determinants of the emergence of parental guilt, such as less time available for raising children and care delegated to third parties due to the need to work outside the home as well as a lack of knowledge on oral health care and the presence of oral health problems [8, 13, 14]. It is believed that many oral health problems can be reduced or even avoided when parents/caregivers have access to information on oral health [15, 16].

Studies on this issue are rare and conducted with non-representative samples of children who require dental care [14, 17]. Parents feel accountable when their children exhibit oral health problems at an early age [14, 17] and the likelihood of feeling guilty increases with the severity of dental caries in children [14]. Knowledge on parental feelings regarding their child’s oral health is important to improving oral health and OHRQoL among children and their families. Such knowledge can contribute to the establishment of public policies aimed at improving oral health care among preschool children [15, 18], especially when considering the low use of oral health services by preschool children in some Brazilian cities [10, 19, 20]. The aim of the present study was to evaluate the influence of children’s oral health problems on parental guilt in the representative, preschool-based sample.

## Methods

### Ethical considerations

The present study received approval from the Human Research Ethics Committee of the State University of Paraíba (Brazil) (process number: 00460133000–11) in compliance with Resolution 196/96 of the Brazilian National Health Council. All participants’ rights were protected. Caregivers read and signed a statement of informed consent prior to the children’s participation.

### Sample characteristics

A cross-sectional study was carried out involving a random sample of 832 male and female children aged three to five years enrolled at private and public preschools in the city of Campina Grande, Brazil. The participants were selected from a total population of 12,705 children in this age group. Campina Grande (population: 386,000) is an industrialized city in northeast Brazil and is divided into six administrative districts. The city has a Human Development Index of 0.72 [21].

The percentage distribution of three-to-five-year-old preschool children in each administrative district was calculated from information provided by the municipal Board of Education. To ensure representativeness, the sample was stratified according to administrative district and type of institution (two-phase sampling method). Preschools were randomly selected from each administrative district in the first phase and preschool children were randomly selected from each preschool in the second phase. Sample distribution was proportional to the total population enrolled in private and public preschools in each administrative district of the city*.* The sample size was calculated based on a 4% margin of error, a 95% confidence level and a correction factor of 1.2 to compensate for the design effect [22]. As the prevalence of parental guilt was unknown, a prevalence rate of 50% was considered to increase the power and because this value gives the largest sample regardless of the actual prevalence [23]. Eighteen of the 127 public preschools and 15 of the 122 private preschools were randomly selected. The minimum sample size was estimated at 720 preschool children, to which an additional 20% was added to compensate for possible losses, giving a total sample of 864 preschool children.

### Eligibility criteria

To be included in the study, the children needed to be between three and five years of age, enrolled in a preschool and free of systemic diseases (based on the reports of the parents/caregivers). Only reports of parents/caregivers were considered for systemic disease; no systemic examination was conducted. The exclusion criteria were the presence of one or more erupted permanent teeth, a history of orthodontic treatment, caregivers not fluent in Brazilian Portuguese and incomplete questionnaires.

### Training and calibration exercise

The training and calibration exercise consisted of two steps (theoretical and clinical). The theoretical step involved a discussion of the criteria for the diagnosis of dental caries and TDI. A specialist in pediatric dentistry (gold standard in this theoretical framework) coordinated this step, instructing three general dentists on how to perform the examination. The clinical step was conducted at a randomly selected preschool that was not part of the main sample. Each dentist examined 50 previously selected children between three to five years of age. Inter-examiner agreement was tested by comparing each examiner with the gold standard (K = 0.85 to 0.90). A seven-day interval was respected between clinical examinations for the determination of intra-examiner agreement (K = 0.85 to 0.90). Data analysis involved Cohen’s Kappa coefficient on a tooth-by-tooth basis. As Kappa coefficients were very good [24], the examiners were considered capable of performing the epidemiological study.

### Pilot study

A pilot study was conducted to test the methodology and comprehension of the questionnaires. The children in the pilot study (n = 40) were not included in the main sample. As there were no misunderstandings regarding the questionnaires or the methodology, no changes to the data collection process were deemed necessary.

### Non-clinical data collection

The collection of the non-clinical data involved one item on the family distress subscale of the Brazilian version of the Early Childhood Oral Health Impact Scale (B-ECOHIS) and questionnaires addressing socio-demographic data, health perceptions and history of toothache. Parents/caregivers were previously contacted to attend a meeting at the preschools, at which they were informed regarding the objectives of the study. Parents/caregivers who agreed to participate signed a statement of informed consent and were then instructed to answer the B-ECOHIS and a questionnaire addressing socio-demographic data. For the B-ECOHIS, the parents/caregivers were instructed to consider the child’s entire lifetime experience of oral health conditions and treatment. All questionnaires were filled out by the parents/caregivers and returned at the end of the meeting.

The B-ECOHIS addresses the perceptions of parents/caregivers regarding the impact of oral health problems on the quality of life of preschool children and their families. This scale is divided into two sections (Child Impact and Family Impact), containing six subscales and thirteen items. Parental guilt was evaluated using the family distress subscale [25, 26]. The item on parental guilt has demonstrated satisfactory internal consistency and reliability [27]. For statistical purposes, parental guilt was dichotomized as absent (only the response option "never") and present (remaining response options: "hardly ever", "sometimes", "often" and "very often"). "Don’t know" responses were not counted [14].

The following socio-demographic data were analyzed: child’s sex, child’s age, parent’s/caregiver’s age, mother’s schooling, type of preschool (public or private) and household income (classified based on the monthly minimum wage in Brazil, which was equal to US$312.50).

Perceptions of the children’s general health and oral health were investigated using the following questions: How would you describe your child’s overall health/oral health status in general? The response options were 1) very good, 2) good, 3) fair, 4) poor and 5) very poor. For statistical purposes, perceptions were dichotomized as good (responses of "very good" and "good") and poor (responses of "fair", "poor" and "very poor") [7].

### Clinical data collection

After the return of the questionnaires and signed statement of informed consent, the clinical exams were performed by three dentists who had undergone the training and calibration exercise. Prior to the exam, the children cleaned their teeth under the supervision of the examiner. For such, each child received a kit containing a toothbrush, toothpaste and dental floss to remove bacterial plaque from the tooth surfaces and facilitate the diagnosis. The children were examined at the preschools in a sitting position in front of the examiner. Lighting was provided by a portable headlamp (Petzl Zoom head lamp, Petzl America, Clearfield, UT, USA). The dentists used individual protection equipment, a sterile mouth mirror (PRISMA®, São Paulo, SP, Brazil), sterile Williams probe (WHO-621, Trinity®, Campo Mourão, PA, Brazil) and dental gauze to dry the teeth.

Dental caries was diagnosed using the International Caries Detection and Assessment System (ICDAS II) [28]. This index has codes ranging from 0 (absence of dental caries) to 6. Due to the epidemiological nature of the present study, code 1 was not used, as drying of the teeth was performed with gauze rather than compressed air. Code 2 is used for white spots and codes equal to or greater than 3 determine different degrees of cavitation. Dental caries was recorded as present when any tooth had a code of ≥ 2 and absent when all teeth received code 0 [28]. Untreated dental caries was also considered in the evaluation of the impact of cavitated lesions in OHRQoL. This variable was categorized as absent/white spot (codes 0 and 2), cavitated anterior teeth (codes ≥ 3), cavitated posterior teeth (codes ≥ 3), cavitated anterior and posterior teeth (codes ≥ 3).

TDI was classified as enamel fracture, enamel + dentin fracture, complicated crown fracture, extrusive luxation, lateral luxation, intrusive luxation and avulsion [29]. A visual evaluation of tooth coloration was also performed. TDI was recorded as present when any type of injury or tooth discoloration was diagnosed. After the exam, a fluoridated varnish was applied to all teeth and children with dental caries or other dental needs were sent for treatment.

### Statistical analysis

**Figure 1:**
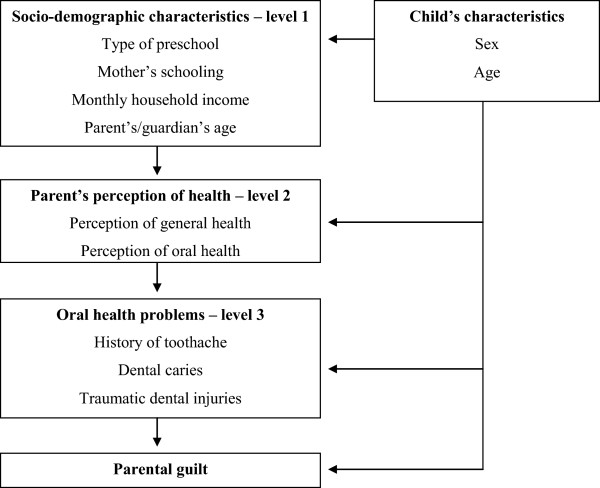
**Analysis model used in the study.**

Descriptive analysis was performed to characterize the sample. Bivariate Poisson regression analysis with robust variance was used to determine the significance of associations between the independent variables and parental guilt (p < 0.05). The multivariate model followed a hierarchical approach from distal to proximal determinants on three levels: [1] socio-demographic data; [2] health perceptions; and [3] oral health problems (Figure 1) [30]. On each level, the backward stepwise method was used for the selection of variables having with a p-value < 0.20 in the bivariate analysis as well as variables considered epidemiological determinants. Variables with a p-value < 0.05 in the adjusted analysis were maintained in the final regression model. Interactions among dental caries and TDI were tested using Wald’s test. Variance inflation factors were calculated to test collinearity among the predictors in the adjusted model. The Statistical Package for Social Sciences (SPSS for Windows, version 20.0, SPSS Inc, Chicago, IL, USA) was used for the statistical analyses.

## Results

A total of 832 pairs of parents/caregivers and their children participated in the present study, corresponding to 96.7% of the total determined by the sample calculation. The loss of 32 pairs was due to a lack of cooperation on the part of the child during the clinical examination (n = 6), incomplete questionnaires (n = 15), absence from preschool on the days scheduled for the examinations (n = 4) and "don’t know" responses on the B-ECOHIS item addressing parental guilt (n = 7). Tables 1 and 2 display the frequency distribution of the socio-demographic data, parental guilt and oral health problems.

**Table 1:** **Frequency distribution of socio-demographic variables**

Variable	Frequency	
	n	%
**Sex**		
Male	430	51.7
Female	402	48.3
**Age**		
3 years	273	32.8
4 years	328	39.4
5 years	231	27.8
**Type of preschool**		
Public	450	54.1
Private	382	45.9
**Mother’s schooling**		
≤ 8 years of study	379	45.7
>8 years of study	450	54.3
**Monthly household income**		
≤ 1 minimum salary	435	54.9
> 1 minimum salary	358	45.1
**Parent’s/guardian’s age**		
≤ 30 years	416	51.1
> 30 years	398	48.9
**TOTAL**	832	100.0

**Table 2:** **Frequency distribution of parental guilt and oral health problems**

Variable	Frequency	
	n	%
**Parental guilt**		
Absent	642	77.2
Present	190	22.8
**History of toothache**		
No	569	68.4
Yes	263	31.6
**Dental caries**		
Absent	279	33.5
Present	553	66.5
**TDI**		
Absent	548	65.9
Present	284	34.1
**TOTAL**	832	100.0

In the bivariate analysis, the following independent variables were associated with parental guilt: age of child, parental perception of child’s general health and oral health as poor, history of toothache, dental caries, cavitated lesions and types of TDI. However, only parental perception of child’s oral health as poor, history of toothache, cavitated lesions and TDI types avulsion/luxation and tooth discoloration remained in the final hierarchical Poisson regression model (Table 3).

**Table 3:** **Hierarchical poisson regression for parental guilt and independent variables among preschool children aged three-to-five years**

Variable	Parental guilt		Bivariate		Multivariate	
	Yes	No	Unadjusted PR*		Adjusted PR†	
	n(%)	n(%)	p-value	(95% CI)	p-value	(95% CI)
**Sex**						
Female	88(21.9)	314(78.1)		1.00	-	-
Male	102(23.7)	328(76.3)	0.530	1.084(0.843-1.392)	-	-
**Age**						
3 years	53(19.4)	220(80.6)		1.00		
4 years	65(19.8)	263(80.2)	0.901	1.021(0.737-1.413)	-	-
5 years	72(31.2)	159(68.8)	0.003	1.605(1.179-2.186)	-	-
**1st level**						
**Type of preschool**						
Private	79(20.7)	303(79.3)		1.00	-	-
Public	111(24.7)	339(75.3)	0.174	1.193(0.925-1.538)	-	-
**Mother’s schooling**						
≤ 8 years of study	98(25.9)	281(74.1)	0.065	1.265(0.985-1.623)	-	-
>8 years of study	92(20.4)	358(79.6)		1.00	-	-
**Monthly household income**						
≤ 1 minimum salary	110(25.3)	325(74.7)	0.128	1.223(0.944-1.586)	-	-
> 1 minimum salary	74(20.7)	284(79.3)		1.00	-	-
**Parent’s/guardian’s age**						
≤ 30 years	101(24.3)	315(75.7)	0.210	1.178(0.911-1.524)	-	-
> 30 years	82(20.6)	316(79.4)		1.00	-	-
**2nd level**						
**Perception of general health**						
Good	136(20.2)	537(79.8)		1.00	-	-
Poor	51(32.9)	104(67.1)	<0.001	1.628(1.243-2.134)	-	-
**Perception of oral health**						
Good	71(12.8)	485(87.2)		1.00		1.00
Poor	119(43.3)	156(56.7)	<0.001	3.389(2.623-4.377)	<0.001	2.010(1.502-2.688)
**3rd level**						
**History of toothache**						
No	72(12.7)	497(87.3)		1.00		1.00
Yes	118(44.9)	145(55.1)	<0.001	3.546(2.750-4.571)	<0.001	2.344(1.755-3.130)
**Dental caries**						
Absent	28(10.0)	251(90.0)		1.00	-	-
Present	162(29.3)	391(70.7)	<0.001	2.919(2.007-4.245)	-	-
**Cavitated lesions**						
Absent/white spots	48(11.0)	389(89.0)		1.00		1.00
Cavitated anterior teeth	12(20.7)	46(79.3)	0.030	1.884(1.065-3.331)	0.302	1.342(0.767-2.348)
Cavitated posterior teeth	63(35.6)	114(64.4)	<0.001	3.240(2.324-4.518)	<0.001	2.002(1.388-2.887)
Cavitated anterior and posterior teeth	67(41.9)	93(58.1)	<0.001	3.812(2.759-5.268)	0.002	1.814(1.247-2.639)
**TDI**						
Absent	126(23.0)	422(77.0)	0.882	1.020(0.783-1.329)	-	-
Present	64(22.5)	220(77.5)		1.00	-	-
**Type of TDI**						
Tooth discoloration	32(33.7)	63(66.3)	0.005	1.567(1.142-2.151)	0.002	1.540(1.169-2.028)
Avulsion/luxation	4(36.4)	7(63.6)	0.195	1.692(0.764-3.746)	0.016	2.029(1.141-3.610)
Enamel + dentin fracture	7(16.7)	35(83.3)	0.471	0.776(0.388-1.548)	0.543	0.780(0.351-1.735)
Enamel fracture or no trauma	147(21.5)	537(78.5)		1.00		1.00

*Unadjusted Poisson regression for independent variables and parental guilt.

†Hierarchical Poisson regression: Level 1 adjusted by characteristics of child and socio-demographic data; Level 2 adjusted by characteristics of child, socio-demographic data and health perception; Level 3 adjusted by characteristics of child, socio-demographic data, health perception and oral health problems (toothache, dental caries and TDI). Variables incorporated into multivariate model (p < 0.20): sex, age, type of preschool, mother’s schooling, monthly household income, perception of general health, perception of oral health, history of toothache, dental caries, untreated dental caries, TDI and type of TDI.

## Discussion

The present study was carried out to investigate the association between parental guilt and oral health problems (dental caries and TDI). The occurrence of toothache, cavitated lesions and type of TDI was found to cause guilt among the parents/caregivers of preschool children. To the best of our knowledge, only two studies have focused on parental guilt in relation to oral health problems [14, 17]. However, the studies cited were conducted with non-representative samples of children who required dental care [14] and were treated under general anesthesia [17]. Others studies have reported only the prevalence of parental guilt using the ECOHIS questionnaire, but failed to analyze associated factors [6, 7, 31, 32]. Moreover, this is the first study to consider the impact of the severity of TDI, different stages of dental caries and the teeth affected in relation to parental guilt.

The frequency of parental guilt in the present sample was 22.8%. This feeling can arise when parents feel accountable for their children’s problems [12] and therefore admit the need to change behavior related to oral health [17]. The literature reports similar rates of parental guilt regarding the oral health problems of children (20.0 to 24.0%) [7, 31, 32]. However, one study found a rate of only 4.2%, which was likely due regular visits to the dentist and consequently lower prevalence of oral health problems [6]. A study of children seeking dental treatment found a higher frequency of parental guilt (35.8%) [14]. These results show that prevalence rates vary across countries/regions as well as in consequence of the age group analyzed, diagnostic criteria employed and type of sample.

The frequency of parental guilt was greater among parents/caregivers who expressed a perception of their child’s oral health as poor. Studies have demonstrated that parental perceptions are associated with clinical characteristics, as children with dental caries are more prone to having their oral health status rated as poor [15, 33, 34]. As parents/caregivers are responsible for their child’s health care and admit that oral health problems can be avoided, the perception of poor oral health may cause feelings of guilt [8, 17]. Moreover, the perception of poor oral health is associated with dental treatment needs in preschool children [15].

The fact that cavitated lesions was associated with parental guilt may be explained by the identification on the part of parents/caregivers of pain symptoms and difficulty eating certain foods [35], as occurs with untreated dental caries on posterior teeth. Although dental health professionals explain the main methods for preventing dental caries [36], many parents fail to put these methods into daily practice [14, 18]. In such cases, parents/caregivers often feel accountable [8, 9] and worry about their child’s future opportunities in life [18]. Parental guilt with regard to cavitated lesions further demonstrates the negative impact of this oral health problem on quality of life [5–7, 35].

TDI, such as avulsion/luxation and tooth discoloration, was associated with parental guilt. Feelings of guilt regarding avulsion/luxation may be attributed to pain, functional limitations and irritability on the part of the child, which are often found in cases of TDI [6]. These injuries can lead to the dislocation of a tooth and pulp involvement, which is a cause of concern for parents/caregivers [18]. TDI can result in tooth discoloration, which exerts an impact on dental esthetics and psychosocial aspects in children [37], with consequent feelings of guilt on the part of parents/caregivers. It should be stressed that, despite the high prevalence rates of dental caries (66.5%) and TDI (34.1%) [2, 4], only those of greater severity were associated with parental guilt, demonstrating that feelings of guilt arise when oral health problems in children are clearly identifiable [17].

A history of toothache was associated with parental guilt, further demonstrating the value of this factor. Previous studies report that this aspect is one of the main reasons for seeking dental treatment in this phase of life [38, 39]. It appears that only oral conditions that cause pain, such as cavitated lesions and type of TDI, are predictors of parental guilt and parents commonly accept the absence of pain in their children to be a sign of good oral health [14]. Indeed, toothache and functional limitations are aspects that exert a greater influence on OHRQoL in preschool children [18, 26, 32]. Thus, parental guilt reflects the impact of oral health problems on OHRQoL, as only severe oral health problems seem to exert an impact on OHRQoL in preschool children due to the presence of toothache and the need for immediate treatment [5, 7, 18, 37].

The present study has the limitations inherent to the cross-sectional design, which pre-empts inferences regarding causality and temporal relationships between variables; thus, longitudinal studies should be conducted to investigate this issue. The possibility of recall bias is also a concern when working with questionnaires. However, measures were taken to minimize this bias, such as the use of a validated questionnaire and the execution of a pilot study. The fact that this study was a preschool-based investigation with a randomly selected, representative sample allows the possibility of extrapolating the findings to the population in the age group analyzed.

## Conclusion

Based on the present findings, parental guilt is mediated by oral health problems of greater severity in preschool children, such as untreated dental caries and type of TDI, due to the association with pain symptoms. The recognition of parental guilt can assist in the drafting of public health policies aimed at reestablishing OHRQoL among preschool children and their families and encouraging changes in behavior toward healthier habits, as parents/caregivers have a direct influence on the oral health of their children.

## Abbreviations

OHRQoL: Oral health-related quality of life
TDI: Traumatic dental injury
ECOHIS: Early childhood oral health impact scale
B-ECOHIS: Brazilian version of the Early Childhood Oral Health Impact Scale
ICDAS: International Caries Detection and Assessment System.
